# Supplementary material for: Human-Robot Joint Misalignment, Physical Interaction, and Gait Kinematic Assessment in Ankle-Foot Orthoses
Source: Sensors (Basel). 2023 Dec 31;24(1):246. doi: 10.3390/s24010246 (PMC10781370; doi:10.3390/s24010246)
Supplement: Supplementary file 1 [file sensors-24-00246-s001.zip › Supplementary_Files_RAndrade_JFigueiredo_PFonseca_JVilasBoas_MSilva_CSantos/Supplementary File S2 - Table S1 Questionnaire Scores.pdf]

Human-Robot Joint Misalignment, Physical Interaction, and Gait Kinematic Assessment in Ankle-Foot Orthoses

Supplementary File S2 – Questionnaire Scores

Table S1. Questionnaire scores per participant and AFO, including average scores and pairwise comparisons.

Subject ID	AFO Model	Q1	Q2	Q3	Q4	Q5	Q6	Q7	Q8	Q9	Q10	Q11	Q12	Q13	CSD-OPUS Score (Max 32)	Total Score (Max 52)
**1**	H2-AFO	2	2	2	2	2	2	2	3	3	3	2	3	3	18	31
	SOF-AFO	2	3	3	3	3	3	3	3	2	3	3	3	3	23	37
	SOL-AFO	2	3	2	3	3	2	3	3	2	3	2	3	3	21	34
**2**	H2-AFO	4	3	3	3	3	4	4	2	3	3	3	3	3	27	41
	SOF-AFO	4	4	1	2	3	4	4	4	1	2	4	3	3	26	39
	SOL-AFO	4	4	4	4	4	4	3	3	3	3	1	3	3	26	43
**3**	H2-AFO	4	3	0	3	3	4	3	4	4	4	4	3	4	28	43
	SOF-AFO	4	4	4	4	3	4	4	4	4	4	4	4	4	31	51
	SOL-AFO	3	3	3	3	3	2	4	3	3	3	4	4	3	26	41
**4**	H2-AFO	3	3	3	3	2	3	3	2	3	3	3	3	3	23	37
	SOF-AFO	4	3	3	3	2	3	3	4	3	3	4	3	3	25	41
	SOL-AFO	3	3	2	2	3	2	3	3	3	3	2	4	3	22	36
**5**	H2-AFO	4	3	3	3	4	4	4	3	3	3	4	4	4	30	46
	SOF-AFO	4	4	3	4	4	4	4	4	4	4	3	4	4	31	50
	SOL-AFO	4	4	4	3	4	4	4	4	4	4	4	3	4	30	50
**6**	H2-AFO	3	2	4	3	3	3	3	4	4	3	3	2	3	23	40
	SOF-AFO	3	4	3	3	3	4	3	4	2	3	4	4	4	27	44
	SOL-AFO	3	4	2	3	3	4	2	4	2	2	4	4	4	25	41
**7**	H2-AFO	2	3	3	3	3	4	4	3	3	3	2	3	4	24	40
	SOF-AFO	3	4	3	3	3	3	4	4	4	4	3	4	4	27	46
	SOL-AFO	2	4	2	3	3	3	3	4	2	3	3	3	2	23	37

**8**	H2-AFO	3	3	3	3	3	3	3	3	3	3	3	3	2	24	38
	SOF-AFO	4	4	4	4	4	4	4	4	4	4	3	3	4	30	50
	SOL-AFO	1	3	1	2	3	1	3	4	2	2	3	4	4	19	33
**9**	H2-AFO	4	1	1	1	1	4	3	1	2	1	3	4	2	21	28
	SOF-AFO	4	3	3	3	3	4	4	3	3	3	4	4	3	29	44
	SOL-AFO	4	3	3	3	3	3	4	3	2	2	3	4	3	26	40
**10**	H2-AFO	4	2	2	2	3	3	4	3	2	2	3	2	2	23	34
	SOF-AFO	4	4	4	4	4	4	4	4	4	4	4	3	4	31	51
	SOL-AFO	4	4	3	3	3	3	4	3	3	3	2	2	3	24	40
**Average**	H2-AFO	3.30	2.50	2.40	2.60	2.70	3.40	3.30	2.80	3.00	2.80	3.00	3.00	3.00	24.10	37.80
	SOF-AFO	3.60	3.70	3.10	3.30	3.20	3.70	3.70	3.80	3.10	3.40	3.60	3.50	3.60	28.00	45.30
	SOL-AFO	3.00	3.50	2.60	2.90	3.20	2.80	3.30	3.40	2.60	2.80	2.80	3.40	3.20	24.20	39.50
**Pairwise**	SOF vs H2	0.30	1.20	0.70	0.70	0.50	0.30	0.40	1.00	0.10	0.60	0.60	0.50	0.60	3.90	7.50
	SOF vs SOL	0.60	0.20	0.50	0.40	0.00	0.90	0.40	0.40	0.50	0.60	0.80	0.10	0.40	3.80	5.80

Subject ID	AFO Model	Q1	Q2	Q3	Q4	Q5	Q6	Q7	Q8	Q9	Q10	Q11	Q12	Q13	CSD-OPUS Score (Max 32)	Total Score (Max 52)
**1**	H2-AFO	2	2	2	2	2	2	2	3	3	3	2	3	3	18	31
	SOF-AFO	2	3	3	3	3	3	3	3	2	3	3	3	3	23	37
	SOL-AFO	2	3	2	3	3	2	3	3	2	3	2	3	3	21	34
**2**	H2-AFO	4	3	3	3	3	4	4	2	3	3	3	3	3	27	41
	SOF-AFO	4	4	1	2	3	4	4	4	1	2	4	3	3	26	39
	SOL-AFO	4	4	4	4	4	4	3	3	3	3	1	3	3	26	43
**3**	H2-AFO	4	3	0	3	3	4	3	4	4	4	4	3	4	28	43
	SOF-AFO	4	4	4	4	3	4	4	4	4	4	4	4	4	31	51
	SOL-AFO	3	3	3	3	3	2	4	3	3	3	4	4	3	26	41
**4**	H2-AFO	3	3	3	3	2	3	3	2	3	3	3	3	3	23	37
	SOF-AFO	4	3	3	3	2	3	3	4	3	3	4	3	3	25	41
	SOL-AFO	3	3	2	2	3	2	3	3	3	3	2	4	3	22	36
**5**	H2-AFO	4	3	3	3	4	4	4	3	3	3	4	4	4	30	46
	SOF-AFO	4	4	3	4	4	4	4	4	4	4	3	4	4	31	50
	SOL-AFO	4	4	4	3	4	4	4	4	4	4	4	3	4	30	50
**6**	H2-AFO	3	2	4	3	3	3	3	4	4	3	3	2	3	23	40
	SOF-AFO	3	4	3	3	3	4	3	4	2	3	4	4	4	27	44
	SOL-AFO	3	4	2	3	3	4	2	4	2	2	4	4	4	25	41
**7**	H2-AFO	2	3	3	3	3	4	4	3	3	3	2	3	4	24	40
	SOF-AFO	3	4	3	3	3	3	4	4	4	4	3	4	4	27	46
	SOL-AFO	2	4	2	3	3	3	3	4	2	3	3	3	2	23	37
**8**	H2-AFO	3	3	3	3	3	3	3	3	3	3	3	3	2	24	38
	SOF-AFO	4	4	4	4	4	4	4	4	4	4	3	3	4	30	50
	SOL-AFO	1	3	1	2	3	1	3	4	2	2	3	4	4	19	33
**9**	H2-AFO	4	1	1	1	1	4	3	1	2	1	3	4	2	21	28
	SOF-AFO	4	3	3	3	3	4	4	3	3	3	4	4	3	29	44
	SOL-AFO	4	3	3	3	3	3	4	3	2	2	3	4	3	26	40
**10**	H2-AFO	4	2	2	2	3	3	4	3	2	2	3	2	2	23	34

	SOF-AFO	4	4	4	4	4	4	4	4	4	4	4	3	4	31	51
	SOL-AFO	4	4	3	3	3	3	4	3	3	3	2	2	3	24	40
**Average**	H2-AFO	3.30	2.50	2.40	2.60	2.70	3.40	3.30	2.80	3.00	2.80	3.00	3.00	3.00	24.10	37.80
	SOF-AFO	3.60	3.70	3.10	3.30	3.20	3.70	3.70	3.80	3.10	3.40	3.60	3.50	3.60	28.00	45.30
	SOL-AFO	3.00	3.50	2.60	2.90	3.20	2.80	3.30	3.40	2.60	2.80	2.80	3.40	3.20	24.20	39.50
**Pairwise**	SOF vs H2	0.30	1.20	0.70	0.70	0.50	0.30	0.40	1.00	0.10	0.60	0.60	0.50	0.60	3.90	7.50
	SOF vs SOL	0.60	0.20	0.50	0.40	0.00	0.90	0.40	0.40	0.50	0.60	0.80	0.10	0.40	3.80	5.80
